# The Pivotal Role of Protein Phosphorylation in the Control of Yeast Central Metabolism

**DOI:** 10.1534/g3.116.037218

**Published:** 2017-02-28

**Authors:** Panayotis Vlastaridis, Athanasios Papakyriakou, Anargyros Chaliotis, Efstratios Stratikos, Stephen G. Oliver, Grigorios D. Amoutzias

**Affiliations:** * Bioinformatics Laboratory, Department of Biochemistry & Biotechnology, University of Thessaly, Biopolis, Larisa 41500, Greece; † National Centre for Scientific Research Demokritos, Agia Paraskevi 15341, Greece; ‡ Cambridge Systems Biology Centre, University of Cambridge, CB2 1GA, UK; § Department of Biochemistry, University of Cambridge, CB2 1GA, UK

**Keywords:** yeast, metabolism, phosphorylation, comparative phosphoproteomics

## Abstract

Protein phosphorylation is the most frequent eukaryotic post-translational modification and can act as either a molecular switch or rheostat for protein functions. The deliberate manipulation of protein phosphorylation has great potential for regulating specific protein functions with surgical precision, rather than the gross effects gained by the over/underexpression or complete deletion of a protein-encoding gene. In order to assess the impact of phosphorylation on central metabolism, and thus its potential for biotechnological and medical exploitation, a compendium of highly confident protein phosphorylation sites (p-sites) for the model organism *Saccharomyces cerevisiae* has been analyzed together with two more datasets from the fungal pathogen *Candida albicans*. Our analysis highlights the global properties of the regulation of yeast central metabolism by protein phosphorylation, where almost half of the enzymes involved are subject to this sort of post-translational modification. These phosphorylated enzymes, compared to the nonphosphorylated ones, are more abundant, regulate more reactions, have more protein–protein interactions, and a higher fraction of them are ubiquitinated. The p-sites of metabolic enzymes are also more conserved than the background p-sites, and hundreds of them have the potential for regulating metabolite production. All this integrated information has allowed us to prioritize thousands of p-sites in terms of their potential phenotypic impact. This multi-source compendium should enable the design of future high-throughput (HTP) mutation studies to identify key molecular switches/rheostats for the manipulation of not only the metabolism of yeast, but also that of many other biotechnologically and medically important fungi and eukaryotes.

Since the advent of the functional genomic technologies, there has been an ongoing community effort to characterize and model the complete metabolic network of the yeast, *Saccharomyces cerevisiae* (Herrgård *et al.* 2008). The aim is to be able to simulate yeast metabolism *in silico* and so generate accurate predictions of the phenotypic consequences of genetic manipulations, including multiple gene deletions (Szappanos *et al.* 2011) and the recruitment of foreign genes to construct novel biosynthetic pathways (Szczebara *et al.* 2003; Galanie *et al.* 2015; Nielsen 2015). Genome-scale stoichiometric models of the yeast metabolic network that allow the computation of the steady-state distribution of metabolic fluxes (Flux Balance Analysis) have proved useful in this regard (Dobson *et al.* 2010; Orth *et al.* 2010).

Despite these successes, there is an urgent need to improve these models by incorporating metabolic control and biomass composition in an accurate and context-dependent manner (Dikicioglu *et al.* 2015), as well as the various levels of transcriptional and post-transcriptional regulation (Pir *et al.* 2012). Several studies have clearly revealed the high importance of post-transcriptional regulation (Gygi *et al.* 1999; Greenbaum *et al.* 2003; Castrillo *et al.* 2007; Schwanhäusser *et al.* 2011). Physiological perturbations can trigger a rapid reconfiguration of the fluxes through the metabolic network and the immediacy of such responses is thought to be largely due to changes at the level of enzyme activity, rather than changes in the expression of enzyme-encoding genes (Ralser *et al.* 2009; Bouwman *et al.* 2011; Oliveira *et al.* 2012; Kochanowski *et al.* 2013). These alterations in enzyme activity are often the consequence of the interactions of these protein catalysts with small molecules, including substrates and cofactors. However, the post-translational modification of enzyme molecules, *e.g.*, by phosphorylation, are likely to play an important role in metabolic adaptations since they also have rapid kinetics (Oliveira *et al.* 2012; Oliveira and Sauer 2012; Schulz *et al.* 2014; Tripodi *et al.* 2015; Chen and Nielsen 2016). Intriguingly, the energetic cost of protein synthesis is nine times higher than that of transcription (Schwanhäusser *et al.* 2011); therefore, post-translational regulation via amino acid modifications seems to be a very rapid and energy efficient level of regulation.

Protein phosphorylation is the most abundant post-translational modification that may alter the structure, function, localization, molecular interactions, or degradation of a protein (Nishi *et al.* 2014), and may therefore function as a molecular switch or rheostat of enzyme activity (Chen and Nielsen 2016). The importance of this level of regulation is highlighted by the fact that up to 23% of intracellular ATP may be utilized by protein kinases for phosphorylating their numerous targets (Ptacek *et al.* 2005; Carpy *et al.* 2014). Furthermore, this type of regulation is expected to be tightly controlled, otherwise the ATP supply would be rapidly depleted (Krebs and Stull 1975). The identification of crucial p-sites in key proteins offers synthetic biologists the prospect of manipulating molecular pathways or organismal phenotypes with greater precision than can be achieved by either the deletion or under/overexpression of complete genes (Oliveira *et al.* 2012; Oliveira and Sauer 2012).

The advent of HTP phosphoproteomic technologies in the last decade has revolutionized the field, since hundreds or even thousands of p-sites may be identified within a single HTP experiment. Nevertheless, serious concerns have been raised about the quality of these p-site identifications in terms of both technical and biological noise (Lienhard 2008); indeed, it has been suggested that up to 65% of these p-sites may be nonfunctional (Landry *et al.* 2009, 2014). In addition, the various phosphoproteomic protocols capture distinct fractions of the total phosphoproteome with moderate overlap among them (Bodenmiller *et al.* 2007). Hence, any analysis of phosphoproteomic data poses a series of challenges (Lee *et al.* 2015; Vlastaridis *et al.* 2016). Thus, before identifying p-sites with potentially significant impact on protein function and organismal phenotype, there is an urgent need to: (i) stringently filter these HTP data and (ii) compile datasets from many and diverse protocols to ameliorate any potential biases (Amoutzias *et al.* 2012).

The goal of this study is to employ a compendium of stringently filtered and diverse phosphoproteomic data from the best-studied model eukaryote, *S. cerevisiae* and the pathogenic fungus *Candida albicans* together with evolutionary, functional genomic, and phenotypic data so as to: (i) reveal the impact of protein phosphorylation on central metabolism, and (ii) prioritize the metabolism-related yeast p-sites in terms of biological significance and assess their potential as targets of future mutation studies with a focus on biotechnological and medical applications. Furthermore, by identifying crucial phosphorylation switches that regulate yeast metabolism, it should be possible, with minimal effort, to significantly improve the predictive accuracy of metabolic flux balance analyses.

## Methods

For *S. cerevisiae*, a high quality compendium of p-sites has been employed from another computational analysis of our group concerning the estimation of the total number of phosphoproteins and p-sites in several eukaryotic species (Vlastaridis *et al.* 2017). This compendium was generated from 20 HTP phosphoproteomic experiments found in 18 publications (Gruhler *et al.* 2005; Chi *et al.* 2007; Li *et al.* 2007; Albuquerque *et al.* 2008; Bodenmiller *et al.* 2008, 2010; Beltrao *et al.* 2009; Huber *et al.* 2009; Holt *et al.* 2009; Gnad *et al.* 2009; Soufi *et al.* 2009; Aguiar *et al.* 2010; Saleem *et al.* 2010; Wu *et al.* 2011; Oliveira *et al.* 2012; Mascaraque *et al.* 2013; Lee *et al.* 2013; Weinert *et al.* 2014). Very stringent criteria were applied, such as 99% correct phosphopeptide identification and 99% correct p-site localization (see Supplemental Material, File S1; spreadsheet: yeast p-sites). This compendium was an update of a previous yeast compendium from 12 HTP datasets (Amoutzias *et al.* 2012). In addition, the PhosphoGRID 2 dataset of manually curated low-throughput (LTP) p-sites (serving as a “gold standard”) (Sadowski *et al.* 2013) was integrated into the compendium. For comparative phosphoproteomic analyses, two datasets from *C. albicans* (Beltrao *et al.* 2009; Willger *et al.* 2015) were mined and filtered by applying the same stringent criteria as for *S. cerevisiae*. All filtered p-sites from the two species are organized in two spreadsheets (*S. cerevisiae* p-sites and *C. abicans* p-sites) within File S1 and File S3.

For the comparative phosphoproteomics analysis between *S. cerevisiae* S288C (Goffeau *et al.* 1996) and *C. albicans* (SC5314 Assembly 21, haploid protein complement), orthologous relationships were retrieved from the *Candida* Gene Order Browser (Maguire *et al.* 2013) using synteny or, if not available, the best Blast hit. To estimate the conservation of yeast p-sites in the orthologs of various ascomycetes, orthologies were retrieved from the fungal orthogroups repository (Byrne and Wolfe 2005; Wapinski *et al.* 2007). For each orthologous pair of sequences, pairwise global alignment was performed with the SSearch software (Pearson 2000) and orthologous amino acids were retrieved from each alignment.

Once orthologs had been identified, the conservation of a p-site in certain ascomycetae ancestors was assessed by two different methods. In the first method, a pairwise comparison of the homologous amino acids between *S. cerevisiae* and another ascomycete was performed. If the amino acid phosphorylated in *S. cerevisiae* was also found conserved as serine, threonine, or tyrosine in the other species, then the p-site was assumed to be present in their common ancestor. In the second method, the ancestral amino acid was inferred by maximum likelihood ancestral sequence reconstruction, using the MEGA7 software (Kumar *et al.* 2016). Conservation of *S. cerevisiae* p-sites in the other ascomycetes is stored in the Excel spreadsheets “conservation_pairwise_comp” and “conservation_MEGA_ASR” of File S3. Divergence dates between extant fungi were retrieved from the TimeTree database (Hedges *et al.* 2015).

For the functional and statistical analyses, many publicly available functional genomics datasets were integrated, such as three protein abundance datasets from two publications (Ghaemmaghami *et al.* 2003; Newman *et al.* 2006), two protein half-lives datasets (Belle *et al.* 2006; Christiano *et al.* 2014), one compendium/list of highly confident essential genes (Giaever *et al.* 2002; Steinmetz *et al.* 2002; Pache *et al.* 2009), one protein ubiquitination dataset (Peng *et al.* 2003), one dataset of highly confident genetic interactions (Costanzo *et al.* 2010), one compendium of highly confident protein–protein interactions (Batada *et al.* 2006), a list of genes and the metabolic reactions that they are involved in (included in the updated version 7.6 of the yeast metabolic model) (Dobson *et al.* 2010), and a dataset of biotechnologically important genes that have been annotated as such in the *Saccharomyces* Genome Database (Cherry *et al.* 2012). The integrated functional data are stored in the Excel spreadsheets “yeast p-sites” and “functional_information” of File S1 and File S2. Of note, many of the above properties/measurements may be context dependent or change significantly from one physiological condition to another.

A negative phosphoproteome was also defined, which comprised an extension of the negative phosphoproteome from our previous study of 2012 (Amoutzias *et al.* 2012). More specifically, in the 2012 study, a nonphosphoproteome comprised 2219 ORFs that had no evidence of phosphorylation, even with less stringent filtering criteria. In this updated analysis, any of these 2219 ORFs that were now found to be phosphorylated were removed from the negative phosphoproteome, resulting in an updated negative set of 2167 ORFs.

Data integration was performed with the PERL programming language and statistical analyses with the R programming language (https://www.R-project.org/) (R Core Team 2015). Mapping of the yeast phosphoregulated enzymes to the KEGG metabolic map was performed with the KEGG mapper computational tool (Kanehisa *et al.* 2012), using the Uniprot identifiers of the yeast phosphorylated proteins.

To control for protein abundance as a potential confounding factor (Levy *et al.* 2012) in the comparison between the phosphoproteome and the negative phosphoproteome, relevant abundance measurements [based on the most thorough dataset of Ghaemmaghami *et al.* (2003)] were converted to log10 values and binned in 8–10 groups. Equal numbers of phosphoproteins and nonphosphoproteins were randomly selected from each bin, thus generating a Protein Abundance Controlled phosphoproteome and negative phosphoproteome. The same procedure was followed for the metabolic phosphoproteome and the metabolic negative phosphoproteome.

For the structural analyses, the available X-ray crystal structures of selected enzymes were retrieved from RCSB PDB (Rose *et al.* 2015). The interactions of p-sites with surrounding residues and ligands or substrates were identified and then all heteroatoms were removed prior to the simulations. Molecular dynamics (MD) were performed for selected enzymes in their native and phosphorylated states with all-atom representation in explicit solvent using AMBER 14 and the ff14SB force field (Case *et al.* 2005; Hornak *et al.* 2006). The phosphorylated enzymes were prepared by mutating the corresponding residues to their phosphorylated forms (net charge of −2e^−^), which were treated with the optimized parameters of the phosaa10 force field (Homeyer *et al.* 2006). Simulations were performed for 100 ns using the GPU-version of the PMEMD program (Salomon-Ferrer *et al.* 2013) and the trajectory analysis was performed with the CPPTRAJ module of AmberTools 15 (Roe and Cheatham 2013) after mass-weighted RMSD fitting with respect to the initial coordinates of the backbone atoms. Visual inspection of the trajectories and rendering of the figures was performed with VMD (v1.9) (Humphrey *et al.* 1996).

### Data availability

The authors state that all data necessary for confirming the conclusions presented in the article are represented fully within the article.

## Results and Discussion

### The updated yeast p-site compendium

The new *S. cerevisiae* compendium consists of 14,339 p-sites in 2633 ORFs (see Table 1 and Excel spreadsheet “yeast p-sites” of File S1) and constitutes a significant increase of 47% (for p-sites) over a previous compendium of 12 publicly available HTP phosphoproteomic datasets (Amoutzias *et al.* 2012). It is designated as 21UHQ, where 21 stands for the number of datasets, U stands for phosphopeptides uniquely matched to only one protein, and HQ stands for high-quality phosphopeptides, based predominantly on 99% correct peptide identification and 99% correct p-site localization. Compared to the original yeast p-site compendium, the new one has been expanded by eight more HTP datasets and also includes the latest version of the PhosphoGRID 2 (PG2) subset (Sadowski *et al.* 2013), which is based on manually curated LTP p-sites. PhosphoGRID is considered the gold standard of yeast p-sites.

**Table 1:** The number of unique p-sites and phosphoproteins identified in the various phosphorylation compendiums and subsets

	Total p-Sites	Total p-Sites Found in PFAM Domains	Highly Confident p-Sites	Highly Confident p-Sites Found in PFAM Domains	Phosphoproteins	Phosphoproteins with Highly Confident p-Sites
12UHQ	9783	2059	2566 (26%)	431	2374	1112 (47%)
20UHQ (only HTP)	13,244	2625	4156 (31%)	698	2587	1421 (55%)
21UHQ (including PG2)	14,339	3036	5519 (38%)	1175	2633	1557 (59%)
21UHQ metabolism (including PG2)	1668	527	499	99	412	197
21UHQ metabolism essential proteins (including PG2)	339	153	79	34	71	36

p-sites identified in three or more experiments are designated as Highly Confident. 12UHQ refers to the Amoutzias *et al.* (2012) dataset. 20UHQ (only HTP) refers to the p-sites identified by 20 HTP experiments in Vlastaridis *et al.* (2017). 21UHQ (including PG2) refers to the p-sites identified by 20 HTP experiments and by PhosphoGrid2 in Vlastaridis *et al.* (2017). 21UHQ metabolism (including PG2) refers to the p-sites identified by 20 HTP experiments and by PhosphoGrid2 in Vlastaridis *et al.* (2017) that were in metabolic proteins. 21UHQ metabolism essential proteins (including PG2) refers to p-sites identified by 20 HTP experiments and by PhosphoGrid2 in Vlastaridis *et al.* (2017) that were in essential metabolic proteins. p-site, phosphorylation site; HTP, high-throughput.

Due to concerns about technical and biological noise in phosphoproteomic data (Lienhard 2008; Landry *et al.* 2009), we constructed a highly confident subset consisting of 5519 p-sites in 1557 ORFs that includes p-sites identified in three or more HTP experiments and/or any of the PG2 LTP data (see Table 1). The criterion for three or more experiments was based on simulations and a series of five different analyses with the original compendium (Amoutzias *et al.* 2012). The corresponding highly confident subset is now designated as 21UHQ_HC, where HC stands for High Confidence.

A crucial issue is the reliability of p-sites that have been identified only once or twice by HTP experiments, since biological and technical noise are serious concerns. In order to address this, the PG2 dataset was employed to perform a crude extrapolation. Of the 536 highly confident p-sites that are detected both by PG2 and any of the 20 HTP experiments, 270 were found in three or more HTP experiments (designated as high overlap HTP), whereas 266 are found in one or two HTP experiments (designated as low overlap HTP). The ratio for high/low overlap is almost one. Therefore, for every high overlap HTP p-site there exists one low overlap HTP p-site that has a high probability of being valid. Based on this empirical ratio, an extrapolation was performed on the whole phosphoproteomic dataset, which has 4156 high overlap and 9088 low overlap HTP p-sites. Thus, we predict that 46% of those 9088 low overlap sites could eventually be verified as highly confident by new experiments. The above crude extrapolation estimates that two thirds (66%) of the current total (low + high overlap) HTP p-sites will turn out to be functional. This is in moderately good agreement with an independent estimation by Landry *et al.* (2009) that was based on other datasets and an evolutionary analysis, where they estimated that 65% of their HTP p-sites could be nonfunctional. Nevertheless, in their dataset, they applied less stringent detection criteria than those we employed and probably included a higher fraction of noisy p-sites.

Our own literature mining revealed (at that time) that the phosphoproteomic data available for fungi other than *S. cerevisiae* were rather limited, although very recently a comparative phosphoproteomic analysis has been performed in 18 fungi (Studer *et al.* 2016). The only other closely related ascomycete for which sufficient phosphoproteomic data were available to allow meaningful comparative analyses was *C. albicans*, with two datasets comprising 9438 nonredundant p-sites. By identifying the homologous amino acids between *S. cerevisiae* and *C. albicans* (see *Methods*), comparative phosphoproteomics revealed that only 7% (692) of those 9438 *C. albicans* p-sites have also been identified as phosphorylated in *S. cerevisiae*. Interestingly, 12% (81/692) of these conserved phosphorylation events between the two species had a mutation from serine to threonine and vice versa to one of the two species. We did not observe such a mutation for phosphorylated tyrosines, most probably due to their very low number (five). These observations are explained by the lack of tyrosine kinases in yeast and the dual specificity of certain serine/threonine kinases that may further phosphorylate some tyrosines (Stern *et al.* 1991; Hunter and Plowman 1997; Zhu *et al.* 2000; Manning *et al.* 2002). Moreover, the use of the 9438 *C. albicans* p-sites together with amino acid conservation in *S. cerevisiae* suggests phosphorylation for another 2122 homologous serines, threonines, and tyrosines in *S. cerevisiae* that have not been detected as phosphorylated yet in that species, but that are likely to be detected by future studies; these would increase the *S. cerevisiae* phosphoproteome by 15%, from 14,399 to 16,461 p-sites.

The fact that few p-sites appear as phosphorylated and conserved between the two species is not surprising. It could be attributed to several factors, such as the incompleteness of the p-site compendia of the two species and experimental biases, since the *C. albicans* compendium was based only on two experiments (Boekhorst *et al.* 2008, 2011). In a recently published study, our group has estimated that the total *S. cerevisiae* phosphoproteome may be *ca*. 40,000 p-sites (Vlastaridis *et al.* 2017). Other contributing factors could be the evolutionary distance of ∼300 million years between *S. cerevisiae* and *C. albicans* (Hedges *et al.* 2015), the high evolutionary turnover generally observed for p-sites, the fast network rewiring at the phosphorylation-regulatory level, and the relaxed localization constraints for p-site conservation (Iakoucheva *et al.* 2004; Moses *et al.* 2007; Landry *et al.* 2009, 2014; Beltrao *et al.* 2009; Shou *et al.* 2011; Freschi *et al.* 2014). Overall, the extrapolation enabled by this comparative phosphoproteomics analysis did not have a profound effect on the quantitative expansion of the *S. cerevisiae* dataset. On the other hand, the conserved p-sites that have withstood such strong forces of mutation and evolution and are detected by these yet imperfect technologies are expected to be of very high functional importance; thus, in qualitative terms, the gain may be greater than the simple increase in p-site numbers implies.

### A substantial part of the yeast central metabolism is regulated by phosphorylation

**Figure 1:**
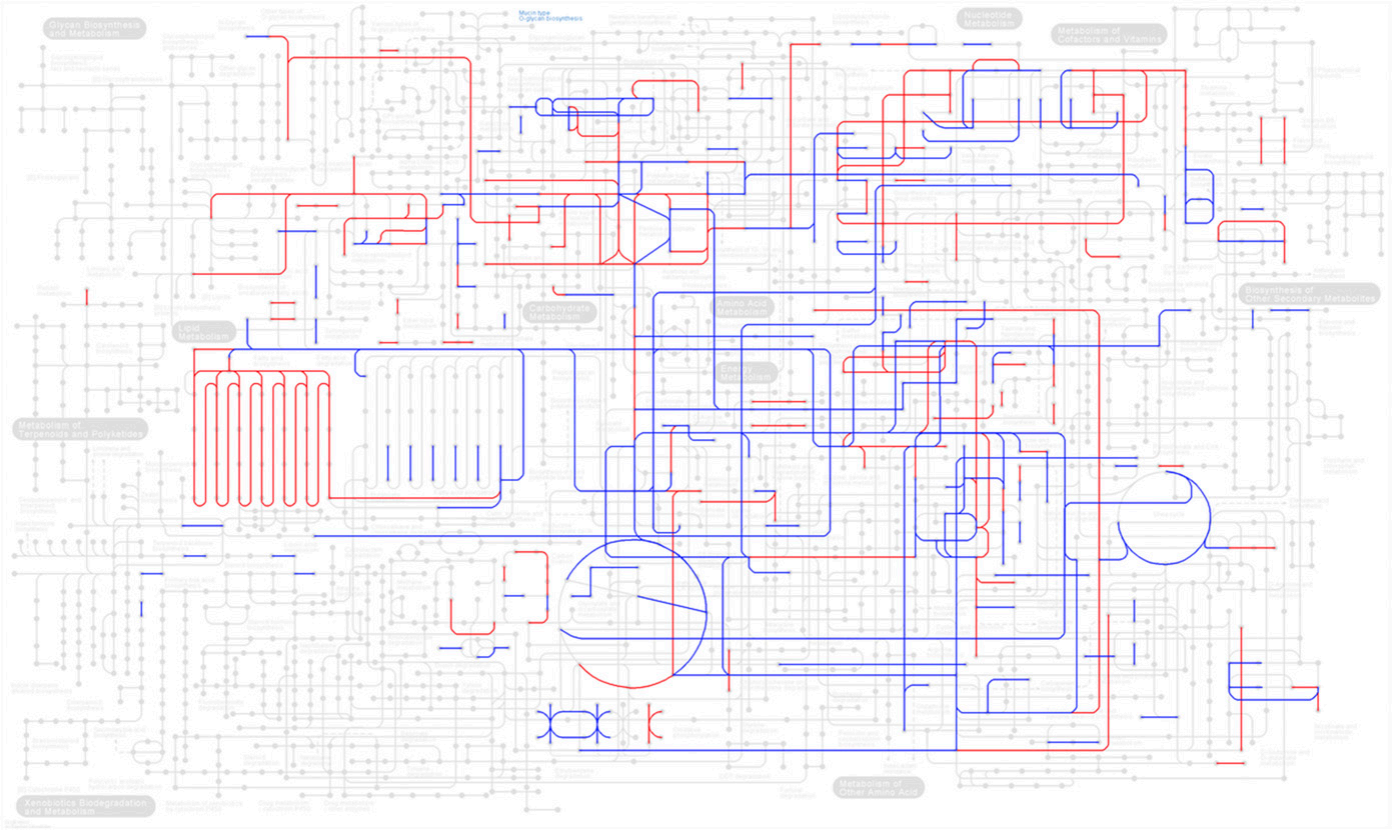
Protein phosphorylation is likely to exert significant control over *S. cerevisiae* central metabolism. Nodes represent metabolites and lines represent reactions in the Kyoto Encyclopedia of Genes and Genomes (KEGG) metabolic map. Blue color is for reactions that are controlled by at least one enzyme that undergoes phosphorylation. Red color is for reactions that are controlled by at least one enzyme that contains High Confidence (HC) p-site/s. Mapping was performed with the KEGG mapper tool (Kanehisa *et al.* 2012), using the Uniprot identifiers of the yeast phosphorylated enzymes.

The Yeast 7.6 genome-scale metabolic model is manually curated by experts and contains 2302 reactions that have been assigned one or more of the 909 (15%) protein-coding genes to be catalyzing these specific reactions. Based on the stringency criteria to define a p-site (designated as ALL for all p-sites and HC), 412 (45%) or 197 (22%) of the metabolic proteins are phosphorylated and may control 1176 or 656 reactions, respectively. Thus, protein phosphorylation is likely to exert significant control over the yeast central metabolism (see Figure 1). A previous analysis on older and less filtered datasets also identified half of the metabolic proteins as being phosphorylated (Oliveira and Sauer 2012). Similarly, a review focused on yeast carbon metabolism reported more than half of the relevant enzymes to be targets of post-translational modifications (Tripodi *et al.* 2015), whereas another review has identified 41 phosphoregulated enzymes that have been experimentally verified (Chen and Nielsen 2016). Furthermore, genetic perturbations of the yeast kinome revealed significant changes in concentrations of hundreds of intracellular metabolites (Schulz *et al.* 2014). Although the current phosphoproteomic data are incomplete in terms of individual p-site detection, an analysis by our group has revealed that most of the phosphoproteins have already been detected (Vlastaridis *et al.* 2017), thus these conclusions appear robust.

A significant proportion of metabolic proteins are phosphorylated and yet there does not seem to be any major enrichment or depletion for phosphorylation in metabolic enzymes compared to the rest of the proteome (45 and 27% for ALL and HC, respectively). Twelve percent (1668/14339) of ALL and 9% (499/5519) of HC p-sites are found in metabolic proteins (designated as phosphometabolic proteins). On average, phosphometabolic proteins have 4 and 2.5 p-sites (ALL and HC, respectively), whereas the rest of the phosphoproteome has 5.7 and 3.7 p-sites, respectively, a statistically significant difference (Wilcoxon p-value < 0.006). In addition, 31% of ALL metabolic and 20% of HC metabolic p-sites are found within PFAM domains, indicating a potentially significant impact on structure, and probably on function. In contrast, 21% of ALL and 21% of HC p-sites are found within PFAM domains (see Table 1). Nevertheless, the next section shows that important enzymes tend to be regulated by phosphorylation.

### The general properties of yeast central metabolism likely to be regulated by phosphorylation

**Figure 2:**
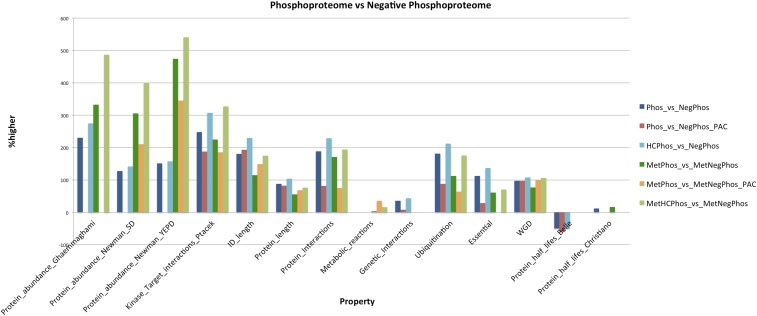
The general properties of the phosphoproteome, compared to the negative phosphoproteome. The bars show which properties of the phosphoproteome are higher/lower (% difference), compared to the negative phosphoproteome. Only statistically significant differences are shown. This is estimated for various datasets. HC, High Confidence subset of the phosphoproteome; MetPhos_vs_MetNegPhos, metabolic proteins of the phosphoproteome *vs.* metabolic proteins of the negative phosphoproteome set. PAC, Protein Abundance Controlled dataset; Phos_vs_NegPhos: phosphoproteome *vs.* negative phosphoproteome.

The general properties of the phosphorylated metabolic proteins (designated as phosphometabolic), compared to the negative phosphometabolic proteins, are summarized in Figure 2 and in more detail in File S2, Excel spreadsheet “stats.” All subsequent reported differences are statistically significant (p-value < 0.05) and were performed with the appropriate Wilcoxon or χ-squared test. Phosphometabolic proteins are: (i) significantly more abundant (305–540% higher), (ii) have more kinase–target interactions (1–1.4 *vs.* 0.3–0.4; 185–327% higher), (iii) have longer total length (602–682 *vs.* 369–388 amino acids; 55–76% higher), (iv) longer intrinsically disordered regions (159–204 *vs.* 71–74 amino acids; 114–175% higher), (v) more protein–protein interactions (1–1.5 *vs.* 0.5; 75–194% higher), and (vi) regulate more reactions (4–5 *vs.* 3.7–3.8; 3–36% higher). Furthermore, a higher fraction of them are ubiquitinated (37–53% *vs.* 19–23%; 64–176% higher). It seems that some synergism exists between protein phosphorylation and ubiquitination in the proteins of the yeast metabolic network (Tripodi *et al.* 2015). All of the above conclusions hold true even when controlling for protein abundance as a confounding factor. GOSlim analysis with Bingo (Maere *et al.* 2005) revealed an enrichment for the GO term “Vacuole,” when phosphometabolic proteins were compared to the background (all metabolic proteins). In general, phosphometabolic proteins retain many of the general properties of the whole phosphoproteome (see Figure 2), except the higher number of genetic interactions, the shorter protein half-lives [only for the Belle *et al.* (2006) dataset; conflicting results for the Christiano *et al.* (2014) dataset], and the higher fraction of essential genes (when controlling for protein abundance). Reassuringly, analyses on this updated whole phosphoproteome compendium confirm the global properties observed in a previous analysis (Amoutzias *et al.* 2012) with a compendium of 12 HTP datasets, even when controlling for protein abundance. This was expected, since the 2012 dataset comprised 2372 phosphoproteins, whereas the new compendium comprises 2633 phosphoproteins. This is another indication of the view that the majority of the yeast phosphoproteome has been discovered (Vlastaridis *et al.* 2017).

In terms of evolution, gene duplications and especially the whole-genome duplication (WGD) that occurred in the hemiascomycete lineage ∼100 million years ago (Pöhlmann and Philippsen 1996; Wolfe and Shields 1997; Kellis *et al.* 2004; Dietrich *et al.* 2004; Scannell *et al.* 2007) are known to have played a significant role in shaping the yeast genome and especially metabolism (Papp *et al.* 2004; Conant and Wolfe 2007; Conant 2014). Intriguingly, a significant fraction of total kinase–substrate relationships may have been rewired during this period by the evolutionary forces of nonfunctionalization, neofunctionalization, and subfunctionalization, suggesting rapid adaption at this level (Amoutzias *et al.* 2010; Freschi *et al.* 2011). On average, 19% (1096/5884) of *S. cerevisiae* protein-coding genes are present in duplicate as a result of the WGD, whereas 23% (207/909) of the genes encoding metabolic proteins are WGD paralogs. For the metabolic enzymes that are phosphoproteins, this proportion increases to 28% (ALL:115/412) and 32% (HC: 64/197). All the above differences are statistically significant (p-value < 0.05), according to the hypergeometric test. This agrees with a previous observation, based on a smaller dataset, that phosphorylation is a factor that affects the survival of genes after WGD (Amoutzias *et al.* 2010).

### Phosphorylation sites of metabolic proteins tend to be more conserved than average

**Figure 3:**
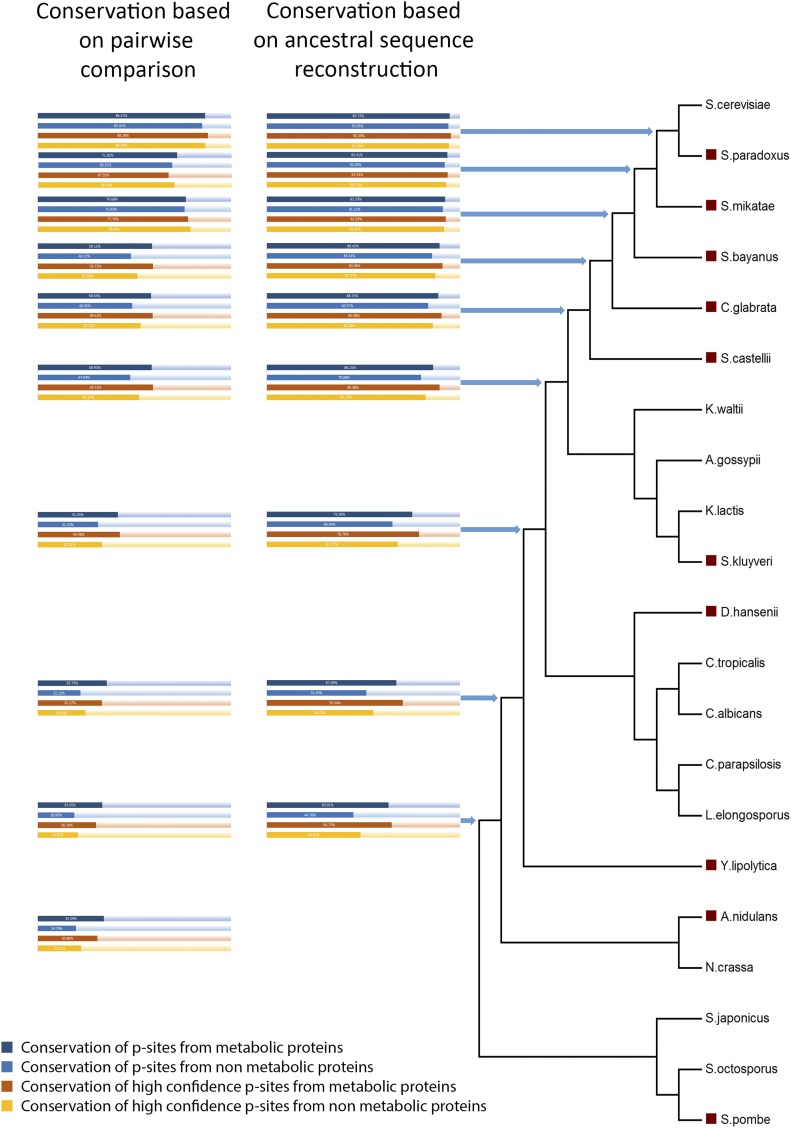
Conservation of p-sites in various ascomycete ancestors. Conservation was inferred by two different methods: pairwise comparison of yeast with other extant species (red boxes in the phylogenetic tree) and ancestral sequence reconstruction. Dark blue bars show % conservation of all metabolic p-sites. Light blue bars show % conservation of all nonmetabolic p-sites. Red bars show % conservation of High Confidence (HC) metabolic p-sites. Orange bars show conservation of HC nonmetabolic p-sites.

Our comparative phosphoproteomic analysis reveals that 115 p-sites, conserved and phosphorylated in both *S. cerevisiae* and *C. albicans*, could regulate 72 metabolic proteins that in turn are involved in 271 reactions of the yeast 7.6 metabolic model (see File S2; spreadsheet “reactions-proteins”). The fraction of conserved phosphorylations between the two species that are involved in metabolism is higher than expected by chance (17%–115/692 *vs.* a background of 12%–1668/14339; hypergeometric test p-value < 2e−5), thus, revealing that p-sites of enzymes tend to be more conserved than the background p-sites. Phosphoproteomic data are not so abundant in other ascomycetes and the observed small overlap may also be attributed to missing data and experimental biases (Boekhorst *et al.* 2008, 2011). We have recently estimated the total yeast phosphoproteome at ∼40,000 p-sites (Vlastaridis *et al.* 2017). To control for this factor, the level of conservation of metabolic protein p-sites in other ascomycetes was also assessed, but only at the amino acid level. Genomic and evolutionary data, together with pairwise comparisons between two extant species or with ancestral sequence reconstruction, were used to infer the homologous amino acid of a yeast p-site in various common ancestors. If the yeast p-site was conserved as serine, threonine, or tyrosine in the inferred ascomycete ancestor, then the assumption was that the phosphorylation event was also present in that ancestor. Next, a comparison of the conservation at the amino acid level was performed for all the p-sites found in *S. cerevisiae* central metabolism *vs.* p-sites in the rest of the proteome (Figure 3). It is clear that the yeast p-sites that are found in metabolic proteins are more conserved than the p-sites in other proteins, and this difference is always statistically significant (Wilcoxon test p-value < 0.05), independent of the method/datasets used. In addition, as the evolutionary distance increases, so does the relative level of conservation of metabolic protein p-sites. Based on the ancestral sequence reconstruction analysis, it is estimated that 1257 budding yeast p-sites identified in 345 ORFs, which in turn are involved in 1003 reactions, could be conserved in the common ancestor between *S. cerevisiae* and *C. albicans*/*Debaryomyces hansenii*.

### Identification of p-sites in proteins that have a biotechnologically interesting phenotype related to metabolism and molecule production

The *Saccharomyces* Genome Database has mined and stored phenotypes caused by various gene perturbations, such gene over/underexpression or even gene deletion. We manually inspected the phenotypes and focused on the ones that, in our opinion, are biotechnologically interesting. These phenotype terms mapped to 850 proteins, of which 408 were phosphoproteins, harboring 2363 p-sites. These phosphoproteins were not all annotated as participating in metabolism. By applying a stringent criterion of HC p-sites situated within conserved domains, we identified 180 of them in 73 phosphoproteins. These findings are summarized in Table 2. Obviously, there exist a significant number of very good candidate p-sites that may regulate biotechnologically important phenotypes, especially those related to increased chemical compound excretion and increased respiratory growth. These candidates should be the initial targets of future studies, *e.g.*, to examine the phenotypic impact of deleting specific p-sites. Due to the inherent technical and biological noise of phosphorylation data, prioritization of p-sites for detailed study is an important task (Beltrao *et al.* 2012; Xiao *et al.* 2016). Readers can perform their own customized prioritization on these data using File S1.

**Table 2:** Number of p-sites that regulate proteins with a biotechnologically interesting phenotype

Phenotype Terms	p-Sites/Proteins (ALL)	p-Sites Within Domains/Proteins (ALL)	p-Sites/Proteins (HC)	p-Sites Within Domains/Proteins (HC)
Chemical compound excretion: increased	1497/248	284/189	564/147	109/43
Fermentative growth: increased	7/3	1/1	2/1	0/0
Fermentative metabolism: increased	85/10	10/6	38/10	3/3
Growth rate in exponential phase: increased	73/8	14/5	38/6	9/2
Nutrient uptake/utilization: increased	124/20	40/8	37/13	13/5
Respiratory growth: increased	416/75	116/41	170/46	43/18
Respiratory metabolism: increased	331/61	70/24	121/38	31/11
Utilization of carbon source: increased	36/8	9/5	16/4	5/2
Vegetative growth: increased	8/5	4/2	0/0	0/0
Viability: increased	67/17	16/9	24/9	2/2
ALL_RELATED_phenotypes	2363/408	496/183	887/247	180/73

p-site, phosphorylation site; ALL, all p-sites; HC, high confidence p-sites.

### Structural simulations of selected phosphorylation sites in two essential metabolic proteins predict a significant impact of phosphorylation on function

**Figure 4:**
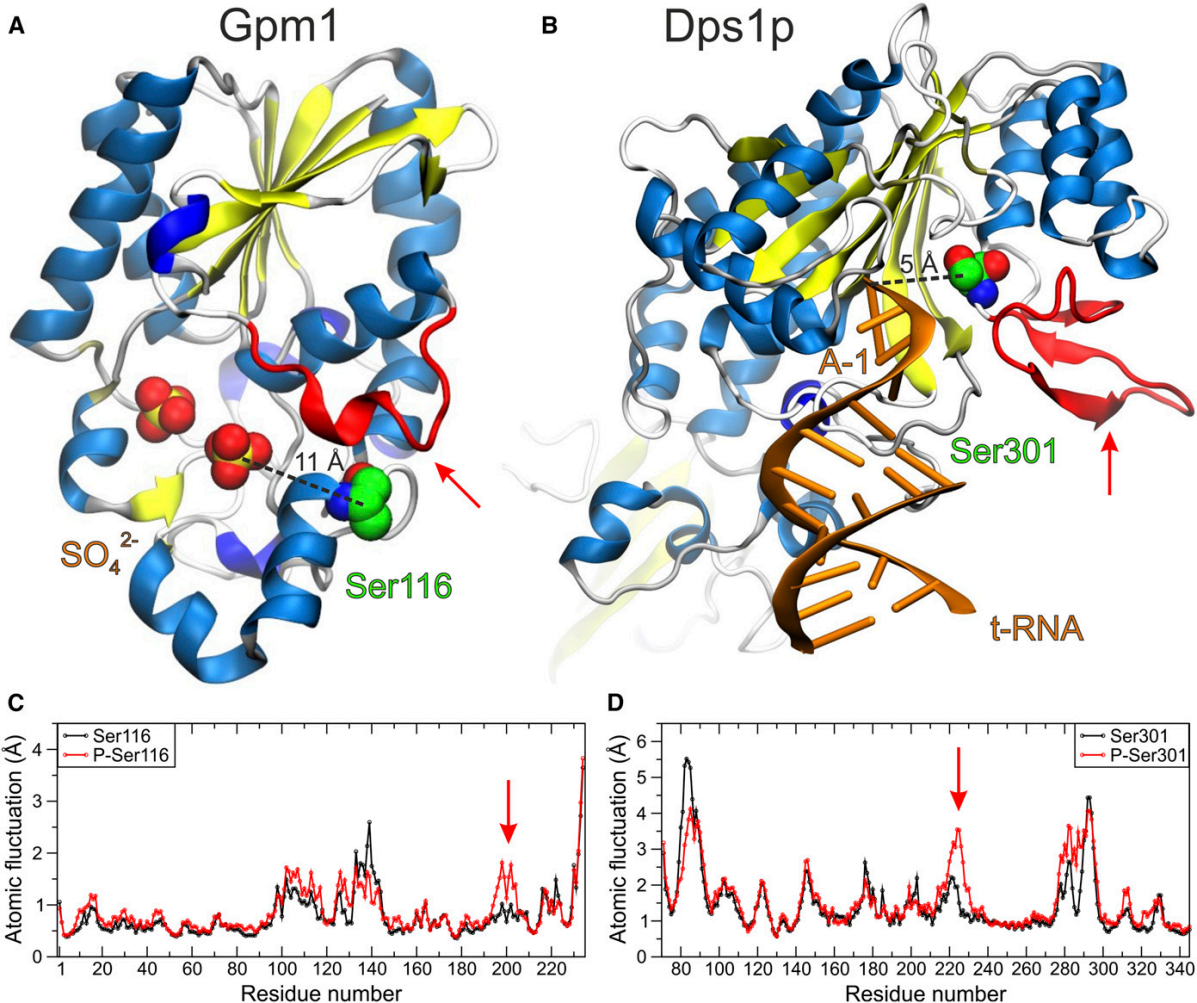
Molecular representations of two p-sites examined with molecular dynamic simulations in (A) the yeast phosphoglycerate mutase (Gpm1p) and (B) aspartyl-tRNA (transfer RNA) synthetase (Dps1p). The X-ray crystal structures of the enzymes are illustrated with cartoons colored by secondary structure and the p-site serine residues are shown with spheres (green C, red O, and blue N atoms). Distances between the p-sites and the catalytic active sites are indicated with dashed lines between Ser116 and a sulfate ion in Gpm1p [ Protein Data Bank identifier (PDB ID): 5pgm], and between Ser301 and adenine-1 (A-1) of tRNA in Dps1p (PDB ID: 1asy). The red arrows indicate regions close to the active sites of the enzymes that display altered dynamics upon phosphorylation. (C and D) Plots of the atomic fluctuations of the backbone Cα atoms extracted from 100-ns MD simulations of the native and phosphorylated enzymes.

Yeast p-sites identified in many experiments, within essential enzymes and also found conserved and phosphorylated in *C. albicans*, could have great potential not only for the manipulation of metabolism (and thus affect the growth rate of *S. cerevisiae*), but also for medical purposes related to other closely related pathogenic fungi. In order to quickly assess the importance of this p-site subset, computational structural analyses were performed on two selected enzymes as a case study. The first enzyme investigated was phosphoglycerate mutase 1 (Gpm1p), which mediates the conversion of 3-phosphoglycerate to 2-phosphoglycerate during glycolysis and the reverse reaction during gluconeogenesis (Heinisch *et al.* 1991). This enzyme has a very promising p-site at Ser116 that was found phosphorylated in 11 HTP experiments. Visual inspection of the crystal structure revealed that this p-site is close to the catalytic site (see Figure 4A). The second protein investigated was aspartyl-tRNA synthetase (Dps1p), an aminoacyl-tRNA synthetase responsible for the charging of tRNA^Asp^ with its cognate amino acid (Sellami *et al.* 1985). Dps1p is a characteristic enzyme of a superfamily that is crucial for the fidelity of translation of the genetic code (Chaliotis *et al.* 2017). Dps1p harbors a very promising p-site at Ser301 that was found phosphorylated in 10 HTP experiments. Examination of the crystal structure of the Dps1p complex with tRNA revealed that this p-site is in direct contact with the substrate (see Figure 4B).

To examine the potential effect of phosphorylation at the enzyme sites described above, we employed a comparative MD study of each enzyme in its native and phosphorylated state. The simulation systems were based on the crystallographic coordinates of the yeast enzymes in the substrate-free forms (PDB IDs: 5pgm for Gpm1p and 1eov for Dps1p) (Rigden *et al.* 1999; Sauter *et al.* 2000). Our simulations indicate that phosphorylation at either Gpm1p-Ser116 or Dps1p-Ser301 can affect substrate binding, either directly or via perturbation of the structural dynamics in regions of the enzymes close to the active site (see Figure 4, C and D). Using their own criteria, readers can use File S1 to prioritize future structural simulations before proceeding to wet lab experiments. With the current datasets, there exist at least 36 p-sites in essential metabolic proteins that have been detected as phosphorylated in both species and need to be investigated with wet lab experiments.

In summary, the integration of HTP data from various genomic, proteomic, functional, and evolutionary sources has highlighted the pivotal role of protein phosphorylation in the control of yeast central metabolism, where almost half of the enzymes involved are phosphorylated. These phosphorylated enzymes, compared to the nonphosphorylated ones, are more abundant, have more protein–protein interactions, regulate more reactions, and a higher fraction of them are ubiquitinated. Furthermore, the p-sites of metabolic proteins are more conserved than the background p-sites. This analysis has also successfully identified and prioritized potential high-confidence p-sites that are likely to have a major impact on enzyme function and should be targets of biotechnological and medical importance. The crucial question in this new era of HTP and integrative science is whether the numerous top-priority targets identified *in silico* will be investigated by LTP validation studies or by highly automated robotic procedures (King *et al.* 2004, 2009).

## Supplementary Material

Supplemental material is available online at www.g3journal.org/lookup/suppl/doi:10.1534/g3.116.037218/-/DC1.
